# Creating a Pro-Regenerative Tissue Microenvironment: Local Control is the Key

**DOI:** 10.3389/fbioe.2021.712685

**Published:** 2021-07-21

**Authors:** Nadya Lumelsky

**Affiliations:** National Institute of Dental and Craniofacial Research (NIDCR), National Institutes of Health (NIH), Bethesda, MD, United States

**Keywords:** pro-regenerative microenvironment, biomaterials, stem cell niche, regenerative medicine, scaffolds

## Introduction

The potential of regenerative medicine to restore tissues and organs compromised or lost due to disease, injury or aging has captured the public imagination and attracted many multidisciplinary researchers to the field. However, despite significant promise, translation to the clinic has so far been modest. This slow progress might at least in part be due to the technical complexity of traditional cell-based approaches, which require *in vitro* manufacturing of large quantities of high-quality clinical grade cells for transplantation, ensuring targeted delivery and survival of these cells *in vivo*, as well as promoting functional morphogenesis of the new tissues and their integration with host tissues. The regulatory path for Food and Drug Administration (FDA) approval of such complex therapies could be arduous. While cell-based therapies remain an important goal for the future of regenerative medicine, an alternative class of therapies—referred to as autotherapies (Lumelsky et al., 2018)—that does not rely on exogenous cell transplantation, but instead attempts to pattern a pro-regenerative tissue microenvironment to optimize endogenous regeneration, might have a simplified regulatory path, and holds significant promise for achieving functional tissue regeneration *in vivo*. While the autotherapies idea is not new (Krzyszczyk et al., 2018; Liu and Segura, 2020; Fetz and Bowlin, 2021), it is particularly important and timely now. Many recent scientific and technical advances, including those in the basic biology of tissue regeneration, single-cell analyses, data science, and bioinformatics, as well as in material science and bioengineering, could propel the field forward and fulfill the therapeutic promise of this class of therapies.

### Multiple Players in the Pro-Regenerative Microenvironment

It is widely recognized that tissue maintenance and regeneration in mammals is controlled by multi-component microenvironmental systems collectively referred to as stem cell niches. In these niches, stem and progenitor cells, various somatic cell types, extracellular matrices (ECMs), and signaling mediators reciprocally interact with each other (Durand et al., 2018; Ruddy and Morshead, 2018). The niches generally remain quiescent under normal adult homeostasis, but injury or disease remodel their regulatory landscapes, and in some cases, induce elements of embryonic or early postnatal regulatory states. This creates a pro-regenerative microenvironment that allows activation, proliferation and differentiation of stem and progenitor cells to build new functional tissues (Nusse et al., 2018; Abbasi et al., 2020; Fuchs and Blau, 2020; Massenet et al., 2021). The regenerative potential of tissues varies widely depending on the species, age, and type of tissue. In some lower vertebrates, new body parts can be completely reconstituted following resection. In mammals, including humans, some tissues, including skin, liver, and oral and intestinal mucosa, regenerate well, while others -such as heart, pancreas, and teeth hardly regenerate at all (Iismaa et al., 2018). Biological age is also a key factor in determining regeneration outcome independent of tissue type, with progressively diminishing regenerative capacity throughout a lifespan (Sousounis et al., 2014).

An extensive body of work has identified numerous biochemical and biophysical mediators, signaling pathways and cell types responsible for control of the pro-regenerative microenvironment in the niche across different species. Many of these mediators and signaling pathways are shared among lower vertebrates and mammals Progress in this field has been greatly facilitated by recent advances in single cell analyses, bioinformatics, and system biology using a variety of organs and animal regeneration models in lower vertebrates, zebrafish and mice, as well as *in vitro* 3-dimensional tissue models such as organoids and tissue chips (Godwin et al., 2013; Mahmoud et al., 2015; Nowoshilow and Tanaka, 2020; Brezitski et al., 2021; Thompson and Takebe, 2021). These studies revealed that regeneration outcomes are determined by a combination of numerous interdependent niche parameters, which precisely align in a spatial and temporal fashion for maintenance and restoration of tissue structure and function. These parameters include the availability and homing of stem and other cell types, including immune cells to the site of injury (Wu et al., 2017; Yin et al., 2017), specific patterns of the ECM remodeling and degradation (Hematti et al., 2018), a timely resolution of acute inflammation and prevention of the onset of chronic inflammation (Kizil et al., 2015), and the establishment of effective vascular supply and neuronal input to the regenerating tissue (Rezza et al., 2014; Mahmoud et al., 2015; Ribatti et al., 2021).

Interestingly, recent results in a variety of model systems strongly suggest that a contribution of the immune system to endogenous tissue regeneration extends beyond its modulation of post-injury inflammation, and also includes direct crosstalk between the innate and adaptive immune systems and the regenerative machinery of the niche to promote or inhibit regeneration (Naik et al., 2018). Failure to achieve a proper patterning of the pro-regenerative microenvironment results in defective regeneration, which manifests itself in tissue fibrosis and scarring instead of productive restoration of functional tissue.

Given the complexity of endogenous tissue regeneration mechanisms, it is not surprising that the outcomes of regenerative medicine therapies in human populations have been variable, as it is now known that they are highly impacted by genetics, age and co-morbidities, including diabetes, obesity and disfunctions of the immune system (Fadini et al., 2020; Masson and Krawetz, 2020). Thus, for the clinical success of regenerative medicine, it will be important to develop and test promising therapies in disease-relevant animal models, including large animal models, that reflect elements of the heterogeneity of human populations (Ribitsch et al., 2020).

### Orchestrating a Pro-Regenerative Microenvironment With Biomaterials

Optimizing endogenous tissue regeneration requires dynamic and predictable spaciotemporal control of the *in vivo* microenvironment. Our current limited ability to achieve such control represents a major roadblock in the field. However, recent progress in biomaterial design lends hope for the future. Over the years, biomaterials employed by regenerative medicine have evolved from inert compounds to powerful effectors of the tissue microenvironment and cellular phenotype. Biomaterials can now be endowed with multiple functionalities that augment tissue regeneration *in vivo*, including: delivery and release of biomolecules with pre-determined kinetics; manipulating stem and progenitor cell lineage commitment, proliferation and differentiation; controlling cellular migration and adhesion; mimicking properties of natural ECMs; exerting local mechanical forces; and modulating immune responses (Wu et al., 2017; Ma and Huang, 2020). Chemists and engineers can now generate predictable and quantitative frameworks for developing new biomaterials with desired functionalities, optimize these functionalities through innovative fabrication approaches and bioassays, and engineer their nanoscale topography, adhesion sites and optimized growth factor presentation to cells (Di Cio and Gautrot, 2016; Vegas et al., 2016; Darnell and Mooney, 2017; Donnelly et al., 2018). Below I offer several examples of promising biomaterial design advances.

Early work showed that biomolecules could be encapsulated into polymeric particles and hydrogels with tunable properties to allow preservation of these biomolecules’ activity for delivery and extended controlled release in tissues. More recent efforts have aimed at designing systems to fit the requirements of a dynamic microenvironment of diseased or injured tissues and release multiple mediators in a spaciotemporal fashion (Hettiaratchi and Shoichet, 2019). For example, in a spinal cord injury (SCI), insufficient axonal growth across an astrocyte scar, which forms as a result of SCI and the absence of growth supporting substrates and cell homing cues, are thought to contribute to failed regeneration. Anderson *et al.* used a multiprong approach in which they treated SCIs in rodents with a combination of viral vectors and hydrogels applied sequentially to the injury site for targeted temporal delivery of multiple growth factors (Anderson et al., 2018). They promoted axon growth with osteopontin, insulin-like growth factor 1 and ciliary-derived neurotrophic factor; induced axon growth supporting substrate with fibroblast growth factor 2 and epidermal growth factor and used glial-derived neurotrophic factor as a homing signal for the growing axons. Providing these three types of stimuli in combination, but not individually, stimulated robust axon regrowth through astrocyte scar borders and across the lesion that was over 100-fold greater than in controls. While this approach is relatively cumbersome and may face obstacles in clinical translation, the study supports the feasibility of endogenous regeneration of complex injuries.

Sophisticated delivery systems in which biomolecule release can be triggered in a combinatorial, sequential or pulsative manner by external stimuli, such as ultrasound, light, temperature, or magnetic/electric fields have also been developed (Cheah et al., 2021; Rapp and DeForest, 2021). Each stimulus has advantages and disadvantages depending on the specific application, but in principle, such approaches provide superior flexibility by assuring tunable and targeted biomolecule release patterns on demand. In the future, it should be possible to couple biomolecule release with biosensing to build feedback systems in which a biomolecule concentration is flexibly adjusted in response to regeneration dynamics.

At this time triggered release studies are primarily conducted in preclinical animal models, and numerous examples of such studies are offered in the excellent recent Reviews referenced above. For example, Zhao et al. used UV light for an on-demand epidermal growth factor release from the hyaluronic acid-based supramolecular hydrogels. Application of this technology to a full thickness rodent skin wound model, resulted in a superior wound healing with respect to granulation tissue formation, and improved angiogenesis compared to controls (Zhao et al., 2020). In another study, Chen et al. used temperature as a trigger for fibroblast growth factor (FGF) release from the chitosan scaffolds incorporating three-dimensionally ordered macroporous particles—inverse opal particles—in a rodent infected skin wound model. The release of the encapsulated FGF from the inverse opal particles was triggered by a high temperature at the inflamed wound site. Because FGF release induced special light reflection changes in the inverse opal particles, the investigators were able to monitor the process in real time. This on-demand release system augmented cell migration and homing at the wound site and ensured the efficient transport of oxygen, nutrients, and metabolic wastes resulting in down-regulation of inflammatory markers, increased collagen deposition and improved granulation tissue formation compared to controls (Chen et al., 2018).

Another promising approach involves building modular chemical frameworks with hydrogels of specifically-designed chemical crosslinker architecture endowing them with precise degradative properties in response to external stimuli, which can be programmed using Boolean logic (Badeau et al., 2018). The authors of this work demonstrated the applicability of their system to the complex spaciotemporal demands of biomolecule delivery by synthesizing 17 distinct hydrogels that collectively yielded all possible YES/OR/AND logic outputs in response to proteases, chemical reducing agents and UV light. The utility of these types of materials for regenerative medicine, is in their potential to pattern tissue microenvironment in response to a combination of exogenous spatiotemporal cues and endogenous cell generated signals. One example of such application is offered by Arakawa et al. who engineered programmable hydrogel photodegradation scheme to generate a customizable vasculature (Arakawa et al., 2017).

In addition to biomolecule delivery, biomaterials also hold promise to improve viral vector-mediated gene delivery by providing more targeted and controllable means for delivering gene cargo to tissues (Wang et al., 2021). These approaches have the potential to overcome the current limitations of uncontrolled virion release and transgene expression as well as undesirable immune response and off-target viral toxicity. Moreover, biomaterial-augmented micro-RNA, small interfering RNA and mRNA delivery approaches show promise for achieving robust and precise control of gene expression. These biomaterial-enabled nucleic acid therapeutics can either induce or inhibit the expression of specific genes and transcription factors involved in control of stem cell niches thereby augmenting endogenous regeneration (Lee et al., 2019; Patel et al., 2019; Yu et al., 2020).

Mechanical forces have been recognized as powerful players in the niche, affecting lineage commitment of stem cells as well as their self-renewal and differentiation during embryonic development and postnatally (Vining and Mooney, 2017; Argentati et al., 2019). Cells in tissues exert internal mechanical forces on their microenvironment—the niche-through adhesive interactions of their cytoskeleton with the ECM and neighboring cells. Reciprocally, external sheer, tensile, and compressive forces act as powerful effectors of niche’s function, cells’ phenotype and ECM properties. Much progress has been made in unravelling molecular mechanisms and signaling pathways associated with internal and external mechanical cues in the niche. These advances provide hope that this knowledge can be harnessed for regenerative medicine applications. Active biomaterials designed for such applications can modulate their physical and chemical properties and transmit mechanical forces *in vivo* in response to a variety of internal and external stimuli. Such biomaterials can be programmed to exert dynamic mechanical forces on cells and tissues in a controllable manner, thereby mimicking the native tissue microenvironment. These biomaterials thus have a capacity to pattern physiological processes, including tissue morphogenesis and regeneration (Özkale et al., 2021).

A strategy capitalizing on mechanical force modulation to promote vascular morphogenesis has recently been described by Wei et al. (2020). In this study, the investigators developed dynamic hydrogel (D-hydrogel) networks in which chemical crosslinks are remodeled in response to traction forces imposed by the cells encapsulated in these hydrogels. The investigators show that the D-hydrogels increased the contractility of human endothelial cells, leading through a series of steps to the activation of focal adhesion kinase, metalloproteinase expression and angiogenesis when the encapsulated cells were transplanted subcutaneously into a mouse. The non-dynamic hydrogels lacking remodeling crosslinks failed to promote angiogenesis. These results suggest that D-hydrogels and other biomaterials that respond to mechanical forces could serve as valuable tools for promoting the endogenous vascular morphogenesis required for successful regeneration of numerous tissues.

In the past, the design of biomaterials was primarily driven by the goals of achieving controlled degradation and superior biocompatibility that would prevent foreign body and fibrotic responses at the site of implantation. These are still important goals, but more recent studies also strive to engineer immunomodulatory biomaterials that control the innate and adaptive immune system responses to pattern a tissue pro-regenerative microenvironment (Chung et al., 2017). This new direction emerged from works demonstrating that the immune system actively participates in tissue regeneration, and that biomaterials can be designed to elicit predictable regenerative responses through the mediation of crosstalk between different immune cells and stem and progenitor cells in the niche (Mariani et al., 2019; Li et al., 2021).

An interesting example of a promising immunomodulatory material was described recently by Griffin et al. (2021). This group developed injectable D-enantiomeric peptide crosslinked Microporous Annealed Particle hydrogels that accelerated the healing of cutaneous wounds and inhibited fibrosis *in vivo*. Importantly, this effect was completely dependent on the generation of an adaptive immune response to the D-enantiomeric peptides, without addition of cells, growth factors or adjuvants. Such immunomodulatory biomaterials, which tip the balance toward regeneration rather than foreign body response and fibrosis, may provide powerful means for achieving minimally invasive endogenous tissue regeneration. Further, predictable modulation of the inflammatory tissue microenvironment can be achieved by delivering extracellular vehicles (EVs) to tissues. It has been shown that EVs derived from mesenchymal stem cells can be used to functionalize biomaterials, to endow them with immunomodulatory and tissue regenerative properties (Brennan et al., 2020).

## Conclusion

Autotherapies have the potential to bring regenerative medicine advances to the clinic. The success of this endeavor, however, is critically dependent on the integration of advances in cellular and molecular regenerative mechanisms with those in bioengineering and material science. Together, these advances could enable robust and predictable control and monitoring of the tissue microenvironment *in vivo.* Significant discoveries have been made in these fields during the last decade, but additional effort is needed to capitalize on these discoveries *via* productive cross-disciplinary collaborations. Given the exquisite complexity of the regenerative mechanisms, multi-prong bioengineering approaches are needed to enable spaciotemporal control of stem cell niches. The new autotherapies will counteract chronic inflammation and tissue destruction and augment regeneration, thereby shifting the diseased tissue homeostasis from fibrosis and scarring to restoration of normal structure and function. Practical application of autotherapies still faces many challenges, but a critical mass of basic science knowledge and technical knowhow is already in place to overcome these challenges and to revolutionize regenerative medicine.
